# *Aspergillus fumigatus ctf1*–a novel zinc finger transcription factor involved in azole resistance

**DOI:** 10.1080/21501203.2024.2342521

**Published:** 2024-04-23

**Authors:** Xiao Gong, Heng Zhang, Wenxu Cheng, Zhangxuan He, Tianyan Ma, Tian Chen, Yi Sun

**Affiliations:** a Department of Dermatology, Jingzhou Hospital Affiliated to Yangtze University, Jingzhou, China; b Department of Otolaryngology, Jingzhou Hospital Affiliated to Yangtze University, Jingzhou, China; c Medical School, Yangtze University, Jingzhou, China

**Keywords:** *Aspergillus fumigatus*, gene knockout, *ctf1*, voriconazole, drug sensitivity, AFUA_1G03800

## Abstract

Elucidating the mechanisms underlying antifungal resistance in *Aspergillus fumigatus*, discovering new antifungal targets, and developing drugs to inhibit resistance are the key approaches to treating *A. fumigatus* infections. Here, we investigated the function of *ctf1* (AFUA_1G03800), a gene encoding a C6 transcription factor. Homologous recombination replacement technology was employed to construct *ctf1-*knockout and revertant strains. Fungal morphological observations revealed that the growth of the knockout strain was slower, showing fewer conidia. The minimum inhibitory concentration of triazoles was determined by performing the E-test and by using the micro-liquid-based dilution method. The results indicated that *ctf1* deletion decreased the susceptibility of *A. fumigatus* to voriconazole by 2-fold. The decreased antifungal sensitivity of *Δctf1* can be attributed to the increased ergosterol content and the overexpression of *mdr1*, *mdr2*, and *mdr4*. Thus, our results on the function of *ctf1* contribute to the elucidation of the mechanisms underlying *A. fumigatus* resistance and the factors associated with *A. fumigatus* virulence.

## 1. Introduction

The fungal pathogen *Aspergillus fumigatus* is ubiquitous in natural ecosystems and urban environments. It is the major cause of opportunistic mould infections in humans (Nywening et al. 2020). In immunocompromised populations, invasive aspergillosis due to *A. fumigatus* can often be fatal, with a high rate of morbidity and mortality (30%–60%) (Douglas et al. 2021) that becomes fatal once the infection spreads to the central nervous system (Fosses Vuong and Waymack 2023). Antifungals are the mainstay of treatment for *A. fumigatus* infections. However, only a few types of antifungal drugs are available as such, including polyenes, echinocandins, and triazoles (Patterson et al. 2016). These antifungal agents have many side effects, a limited spectrum of activity, fewer targets, a potential to form fungal biofilms, and susceptibility to fungal resistance, which hampers the effectiveness of the antifungal therapy (Fuentefria et al. 2018). Therefore, elucidating the mechanisms associated with drug resistance of *A. fumigatus*, developing drugs to counter antifungal resistance, and identifying new antifungal targets can help treat diseases caused by *A. fumigatus*.

Zinc cluster proteins (ZCPs) constitute a large family of zinc-binding proteins that are found only in the fungal community with Zn(II)_2_Cys_6_ dinuclear cluster DNA-binding domains (DBDs). A majority of ZCPs are unique, with specific transcription factors as they possess only a single zinc finger unit bound to two zinc atoms, which enables them to interact with DNA as monomers, as homodimers, or as heterodimers (MacPherson et al. 2006). ZCPs also possess regulatory and activation structural domains along with the DBD. The regulatory structural domain contains an integral region called the middle homology region (MHR). Although it possesses low homology among ZCP members and not all ZCPs have MHR, this region spans approximately 80 amino acids and is believed to regulate the transcriptional activity of these factors (Schjerling and Holmberg 1996). For instance, it participates in the partial deletion of Pdr1 and Pdr3 in the MHR, making it a constitutive activator (Kolaczkowska and Goffeau 1999). The acidic region of ZCP located at the C-terminus is the activation structural domain. It is functionally and structurally diverse and less conserved (Schjerling and Holmberg 1996). Pdr1 and Pdr3 also have gain-of-function mutations in this region (Kolaczkowska and Goffeau 1999). Additionally, the activation structural domain can regulate the function of ZCP via phosphorylation modification or changes in the autoinhibitory conformation (Zhou and Kohlhaw 1990; Sadowski et al. 1996).

Previous studies have demonstrated that ZCP exhibits functional diversity. In yeast, Gal4 and ArgRII regulate the expression of galactose-metabolising enzyme genes and arginine metabolism, respectively (Akache et al. 2001). In *Aspergillus oryzae*, MalR controls the expression of maltose-using cluster genes and the production of amylolytic enzymes (Konno et al. 2018). Upc2 and Ecm22 are the yeast sterol regulatory element-binding proteins that regulate the transcription of sterol biosynthesis genes (Vik and Rine 2001). AflR plays a role in the aflatoxin synthesis pathway in several toxin-producing fungi and regulates the expression of several genes for toxin synthesis (Caceres et al. 2020; Khan et al. 2021). ZcfA is essential for constitutively maintaining the balance between anaphaseic and sexual development in *Aspergillus*, such as *Aspergillus flavus* (Son et al. 2020). Pcz1 controls *Penicillium* growth and conidial germination (Gil-Durán et al. 2015). PacC mediates at least three regulatory pathways that help *Aspergillus constructus* grow under alkaline pH or saline stress (Picazo et al. 2020). Pdr1 confers drug resistance to *Penicillium* by regulating the expression of drug efflux pump genes, such as ATP-binding cassette (ABC) transporter proteins *cdr1, pdh1*, and *snq2* (Klimova et al. 2014). Thus, ZCP is involved in regulating several life processes, including primary metabolism, secondary metabolism, growth and reproduction, and stress response of fungi. It plays a crucial role in fungal drug-resistance mechanisms. Nevertheless, a few studies have investigated the function of ZCP in *A*. *fumigatus*.

The role of efflux pumps in *A. fumigatus* antifungal resistance has been widely studied. A previous study reported that the synthetic activation of the zinc cluster transcription factor Mrr2 in *Candida albicans* upregulated the expression of the gene encoding the Cdr1 efflux pump, which increased fluconazole resistance in *Candida albicans* (Schillig and Morschhäuser 2013). Based on this study, we hypothesised that a similar Mrr2 is present in *A. fumigatus*, which may play a crucial role in azole resistance. Homology comparison revealed that such a gene (GenBank: AFUA_1G03800, *C6 transcription factor*, putative) is present in the genome of *A. fumigatus*. We termed this gene *C6 transcription factor 1* (*ctf1*) and investigated its role in *A. fumigatus*.

## 2. Materials and methods

### 2.1. Fungal strains and plasmids

The pyrimidine-deficient strain A1160 (*ΔKU80* and *pyrG*-) (purchased from http://www.fgsc.net/asperg.html) was used as the host fungi for the transformation of *A. fumigatus*. This strain could not grow on uracil-deficient media. The genetic defect of *ΔKU80* increased the probability of homologous recombination. The wild strain WT (*ΔKU80, pyrG+*) was constructed in our laboratory by randomly introducing the *pyrG* into A1160 and was used as the experimental control. *A. flavus* ATCC 203204 was used as the quality control strain for antifungal susceptibility tests. *pyrG* was obtained from plasmid pLAX223 and used for screening markers. The hygromycin B-resistant gene *hph* and fluorescent marker gene *egfp* were obtained from plasmid pCT74.

### 2.2. Preparation of drugs

ITR, VOR, and POS drug powders were purchased from Shanghai Aladdin Biochemical Technology Co. (Shanghai, China). The antifungal drugs were dissolved in dimethyl sulphoxide (Tianjin Yongda Chemical Reagent Co., Ltd., Tianjin, China) to prepare the working solution of 6,400 μg/mL. Hygromycin B (Shanghai Yeasen Biotechnology Co., Ltd., Shanghai, China) was dissolved in distilled water to prepare a working solution of 100 mg/mL. The prepared drugs were filtered into sterile EP tubes using 0.22-μm needle filters (Shanghai Yeasen Biotechnology Co., Ltd., Shanghai, China).

### 2.3. *Construction of* Δctf1 *and* Δctf1∷ctf1+

#### 2.3.1. Construction of a triple-fusion knockout box

The PCR primers used in this experiment are shown in the S1 file. The primer design method was referred from Zhao et al. (2019). First, the genomic DNA of WT was extracted (MolPure® Fungal DNA Kit Fungal DNA Extraction Kit, 18812ES, Shanghai Yeasen Biotechnology Co., Ltd., Shanghai, China) and PCR was performed to amplify the flanking sequences upstream of the coding region of the *ctf1* by using the primers P1 and P2 and downstream of the coding region of the *ctf1* by using the primers P3 and P4. Then, plasmid pLAX223 was used as a DNA template (MolPure® Plasmid Mini Kit Plasmid Small Extraction Kit, 19001ES, Shanghai Yeasen Biotechnology Co., Ltd., Shanghai, China), and the *pyrG* fragment was amplified with primers pyrG-n-F and pyrG-n-R. All of the above-mentioned PCR products were verified by 1% agarose gel electrophoresis and product purification (MolPure® PCR Purification Kit PCR Product Purification Kit, 19106ES, Shanghai Yeasen Biotechnology Co., Ltd., Shanghai, China) before proceeding to the next step of the experiment. Finally, the abovementioned three gene sequences were fused by PCR with primers P5 and P6. The PCR products were verified by 1% agarose gel electrophoresis.

#### 2.3.2. Protoplast transformation and validation of the knockout strain

Based on the method described by Zhao et al. (2019), the *ctf1-*knockout cassette was introduced into protoplasts prepared from *A. fumigatus* A1160. Then, it was evenly coated on a uracil-free CZA solid medium, incubated at room temperature for 24 h, and placed in a 37 °C warm box, and continued to incubate for 3–5 days. Fungal DNA was extracted from the positive transformants after 2 passages. PCR amplification was performed using the validation primers Awm-F1 and Awm-F2 with P4. After the products were verified by 1% agarose gel electrophoresis, the PCR products were sent to Sangon Biotech (Shanghai) Co., Ltd. (Shanghai, China) for sequencing to verify whether the knockdown was successful.

#### 2.3.3. Construction and validation of revertant strains

Based on the method reported by Tan et al. (2023), the *ctf1* fragment was amplified by PCR using WT DNA as a template with primers ctf1-F and ctf1-R and verified by 1% agarose gel electrophoresis. The pCT74 plasmid DNA was used as a template and digested using restriction endonuclease KpnI and restriction endonuclease NaeI. After 1% agarose gel electrophoresis of the obtained double-enzymatic plasmid, the gene fragment corresponding to a length of 3,000 bp was excised and purified to obtain a fragment with a thiamphenicol resistance gene (*hph*) and a fluorescent marker gene (*egfp*). The *ctf1* fragment was genetically recombined *in vitro* with the double-enzymatic plasmid gene fragment (Hieff Clone® Plus One Step Cloning Kit, 10911ES, Shanghai Yeasen Biotechnology Co., Ltd., Shanghai, China) and transformed using DH5α Chemically Competent Cells (11802ES). The transformed cells were then evenly coated on LB+Thymidine B screening medium and cultured in a light-avoiding environment at 35 °C for 24–48 h. The recombinant plasmid DNA was extracted and PCR amplification was carried out with the replica plasmid validation primers ctf1-yz-F and ctf1-yz-R, and the recombinant plasmid PCR products were purified after the products displayed the target bands by 1% agarose gel electrophoresis. The recombinant plasmid containing the *ctf1* fragment was introduced into protoplasts prepared with the knockout strain and transformed into an SDA medium containing thymidine B. The DNA of the transformants was extracted and sent to Sangon Biotech (Shanghai) Co., Ltd. For sequencing, the inclusion of *hph* in the results was considered to indicate a successful *Δctf1∷ctf1+*.

### 2.4. Morphological observation of fungi

Notably, 1 μL of conidial suspension (concentration 1 × 10^6^ spores/mL) was spotted in a CZA Petri dish and incubated at 37 °C for 48 h. The diameter of individual colonies was measured. The spores of the abovementioned colonies were collected in sterile distilled water and diluted 1,000 times in multiplicity, after which the numbers of spores were counted under the microscope using a haematocrit plate. This experiment was repeated thrice. The cultured *Aspergillus* was uniformly spread onto slides and stained by adding a drop of fungal fluorescent stain (Jiangsu Lifetime Biological Technology Co., Ltd., Taizhou, China). Then, a fluorescence microscope was used to observe the morphological characteristics of the fungus.

### 2.5. E-test for drug sensitivity

ITR (Liofilchem, ICZ 92148), VOR (Mérieux, VRC 532800), and POS (Liofilchem, PCZ 92152) strips were used in this experiment. The results were obtained by incubating the Petri dishes in a 35 °C incubator for 48 h (Szekely et al. 1999).

### 2.6. Microdilution-based antifungal susceptibility

The micro-liquid-based dilution method based on the CLSI M38-A2 (Clinical and Laboratory Standards Institute 2017) was applied. The working concentrations of antifungal drugs were as follows: 8–0.0625 μg/mL for ITR and VOR and 4–0.03 μg/mL for POS. The plates containing conidial suspensions were incubated at 35 °C for 48 h. The lowest drug concentration that inhibited 100% of the fungal growth at the end of the incubation period was considered the minimum inhibitory concentration (MIC). The experiment was performed in triplicate.

### 2.7. Transcriptome sample preparation and RNA-seq analysis

Mycelium and spores of *A. fumigatus* were collected at a wet weight of 50 mg after 72 h of incubation at 37 °C in an SDA solid medium. Total fungal RNA was extracted based on the instructions for the TRlzol reagent (Life Technologies, South San Francisco, CA, USA). The NanoDrop 2000 (Thermo Fisher Scientific, Wilmington, DE, USA) was used to detect the RNA concentration and purity. RNA integrity was determined using the RNA Nano 2100 assay kit in the Agilent Bioanalyzer 6000 system (Agilent Technologies, Santa Clara, CA, USA). Poly-T oligonucleotide-attached magnetic beads were used to purify mRNA from total RNA. Double-stranded cDNA was synthesised, and DNA fragments were adenylated at the 3’ end and then attached to NEBNext articulators with hairpin loop structures for hybridisation. Library fragments were purified using the AMPure XP system (Beckman Coulter, Danvers, MA, USA). cDNA was ligated using 3 μL of USER Enzyme (NEB, Ipswich, MA, USA) with size-selected junctions for 15 min at 37 °C, followed by 95 min at 5 °C before performing PCR. PCR was performed using Phusion high-fidelity DNA polymerase, universal PCR primers, and index (X) primers. Finally, the PCR products were purified using an Agilent Bioanalyzer 2100 system (AMPure XP system), and the library quality was evaluated. The libraries were sequenced using the Illumina NovaSeq platform as per the manufacturer’s protocol to generate 150-bp paired-end reads. The experiment was repeated thrice. The bioinformatics pipeline tool BMKCloud (www.biocloud.net) online platform was used to further process the raw reads.

### 2.8. Mapping and normalizing the gene expression for reading reference genomes

Reads containing adapters, reads containing ploy-N, and low-quality reads from the raw data were excluded to obtain clean data. The contents of Q20, Q30, and GC and the sequence repeat levels were calculated for the clean data. The reference genome was mapped using the Hisat2 tool software. The transcript abundance was normalised using the transcript fragment number per kilobase per million mapped fragments (FPKM) metric to screen DEGs among different treatments. Differential expression analysis was performed for different conditions/groups using DESeq2_edgeR. A fold change of ≥1.5 with *p* < 0.01 was set as the differential gene screening criteria. The statistical enrichment of DEGs in the KEGG pathway KOBAS database and clusterProfiler software were used to test.

### 2.9. Determination of ergosterol content

Based on the method given by Breivik and Owades (1957), the fungal sterols were extracted using n-heptane and diluted with anhydrous ethanol, which was then subjected to full-spectral scanning using Nanodrop One (Thermo Fisher Scientific, Beijing, China). The absorbance was detected at 240 nm to 300 nm and the absorbance curve was constructed. The experiment was performed in triplicate. The ergosterol content was calculated using the following absolute quantitative formulae:

$$\mathrm{Percentage}\;\mathrm{of}\;\mathrm{ergosterol}\;\mathrm{content}\;\left(\%\right)=\left\{\left[\left(A281.5/290\right)\;\times F]/\mathrm{wet}\;\mathrm{weight}\text{\textbackslash{}break}\;\;\;\;\;\;\;\;\;\;\;\;\;\;\;\;\;\;\;\;\;\;\;\;\;\;\;-[\left(A230/518\right)\;\times F\right]/\mathrm{wet}\;\mathrm{weight}\right\}\;\times \;100\%$$

F: Dilution of sterol extracts in anhydrous ethanol; A281.5: Absorbance of the final extract at 281.5 nm UV; A230: Absorbance of the final extract at 230 nm UV.

### 2.10. qPCR analysis of gene expression differences

Mycelium and spores of *A. fumigatus* were collected at a wet weight of 50 mg after 72 h of incubation at 37 °C in an SDA solid medium. Fungal total RNA was extracted as per the instruction manual of the TRlzol reagent. The concentration and purity of total extracted RNA were determined using NanoDrop One. The cDNA was obtained by performing reverse transcription using Hifair® III 1st Strand cDNA Synthesis SuperMix (Shanghai Yeasen Biotechnology Co., Ltd., Shanghai, China). The concentration and purity of the cDNA were measured using NanoDrop One. The target genes were *mdr1*, *mdr2*, *mdr3*, *mdr4*, *cyp51A*, *cyp51B*, and *mfs56*. The results were normalised to the *actin* expressions. The primer sequences are shown in the S2 file. Fluorescent labelling was performed using Hieff® qPCR SYBR® Green Master Mix (No Rox). The Ct values were determined using a qPCR instrument (Suzhou Molarray Biological Technology Co., Ltd., MA-6000, Suzhou, China). The experiments were performed in triplicate.

### 2.11. Statistical analysis

The GraphPad Prism8 software was used for data analysis and plotting graphs. Data were expressed as the mean ± standard deviation using a one-way analysis of variance (ANOVA). A comparison of means between two groups was performed using the student’s *t*-test. The 2^−ΔΔCT^ method was used to analyse the qPCR data. *p* < 0.05 for experimental results was considered statistically significant.

## 3. Results

### 3.1. *Bioinformatics analysis of* ctf1 *in* A. fumigatus

**Figure 1.**
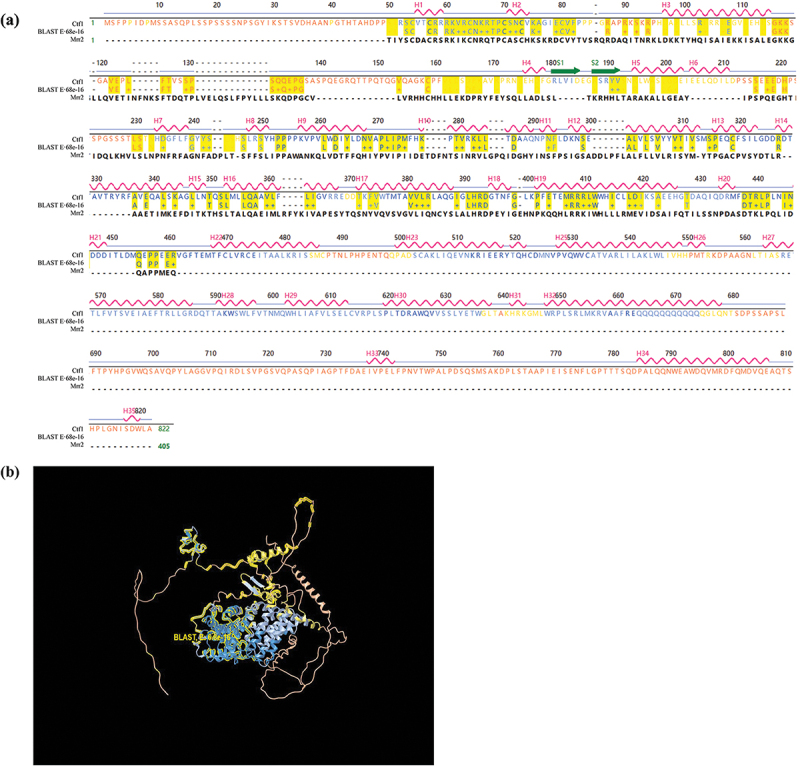
Structural comparison of Ctf1 and Mrr2. (a) Primary and secondary structure of Ctf1 and comparison of the amino acid sequences of Ctf1 with Mrr2. Ctf1 consists of 822 amino acids with 35 α-helices (H1-H35) and 2 β-folds (S1 and S2); Mrr2 consists of 405 amino acids; BLAST, E:6.8e-16 indicates the homologous sequence. (b) Protein structural modelling of Ctf1. The centre is a highly conserved zinc finger structure containing an intermediate homology region on the outside. Bold yellow lines (BLAST, E:6.8e-16) indicate the homologous sequence.

We found that a C6 transcription factor (XP_750130.1) in *A. fumigatus* has the highest homology (57%) to Mrr2 by protein BLAST comparison in the NCBI website. Structurally, it consists of 822 amino acids with 35 α-helical and 2 β-folded (Figure 1(a)), containing two highly conserved structural domains, a fungal transcription factor regulatory immediate homology region, and a GAL4-like Zn(II)_2_Cys_6_ (or C_6_ zinc) binuclear cluster DNA-binding domain. The model of structural similarity between Ctf1 and Mrr2 is depicted in Figure 1(b). In addition, we found the gene (AFUA_1G03800) encoding this transcription factor and called it *ctf1*. This gene contains 2,524 base pairs and 2 exons and is located on chromosome 1.

### 3.2. *Effect of* ctf1 *on the growth of* A. fumigatus

**Figure 2.**
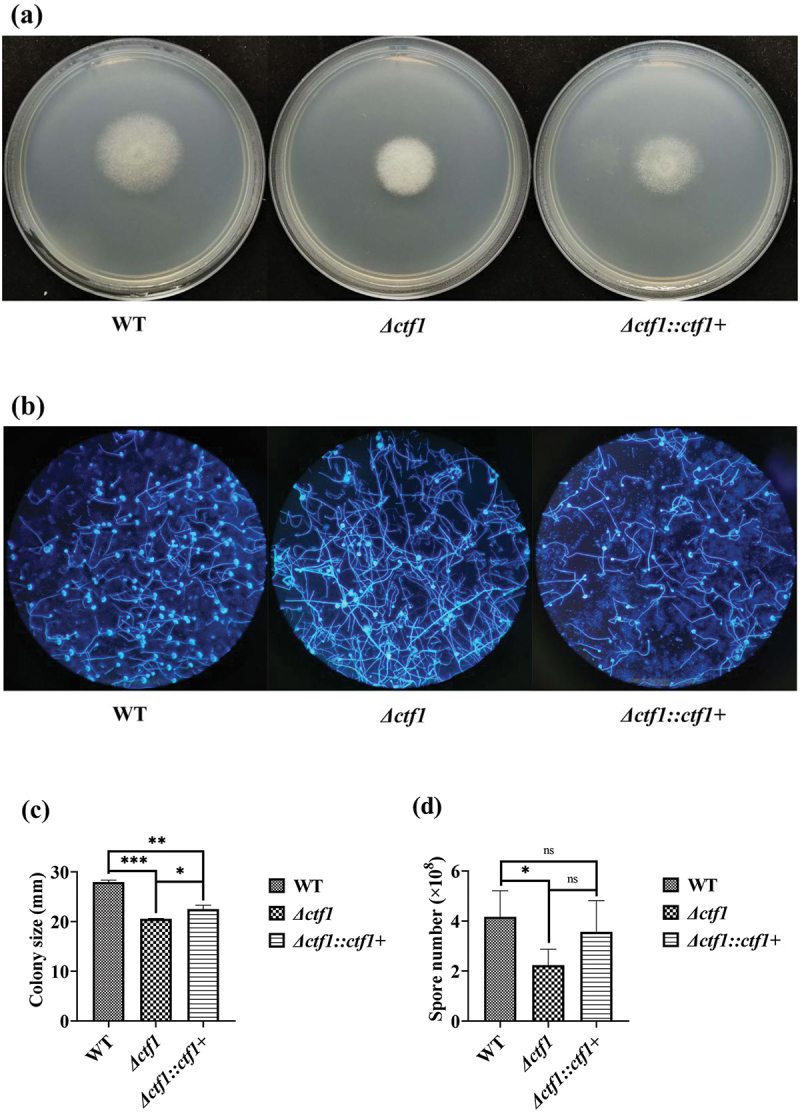
Growth of the strains. (a) Colonies in CZA dishes after incubation at 37 °C for 48 h. (b) Morphology of the strains observed under the microscope after fluorescent staining (×100). (c) Comparison of colony diameters after 48 h of incubation at 37 °C in CZA dishes. (d) Comparison of spore numbers after 48 h of incubation at 37 °C in CZA dishes. ***p < 0.001; **p < 0.01; *p < 0.05; ns, *p* > 0.05.

*A*. *fumigatus* demonstrated morphological changes after the *ctf1* knockout. In the CZA medium, colonies of *Δctf1* exhibited denser fluff hyphae relative to wild-type (WT) (Figure 2(a)). Under the microscope, the number of conidial heads of the knockout strain was significantly reduced when compared to WT, and the mycelium was longer (Figure 2(b)). The colony diameter of *Δctf1* was significantly reduced (*p* < 0.001; Figure 2(c)) and the number of spores was significantly decreased (*p* < 0.05; Figure 2(d)) when compared with that of WT. These changes in growth were partially restored by reintroducing *ctf1* into the *Δctf1* strain, suggesting that mutations in *ctf1* inhibited the growth of *A. fumigatus*. As the reintroduced *ctf1* is not necessarily in its original position, the phenotypic changes it causes in the strain are not always fully recovered.

### 3.3. *Role of* ctf1 *in the sensitivity of* A. fumigatus *toward azoles*

**Figure 3.**
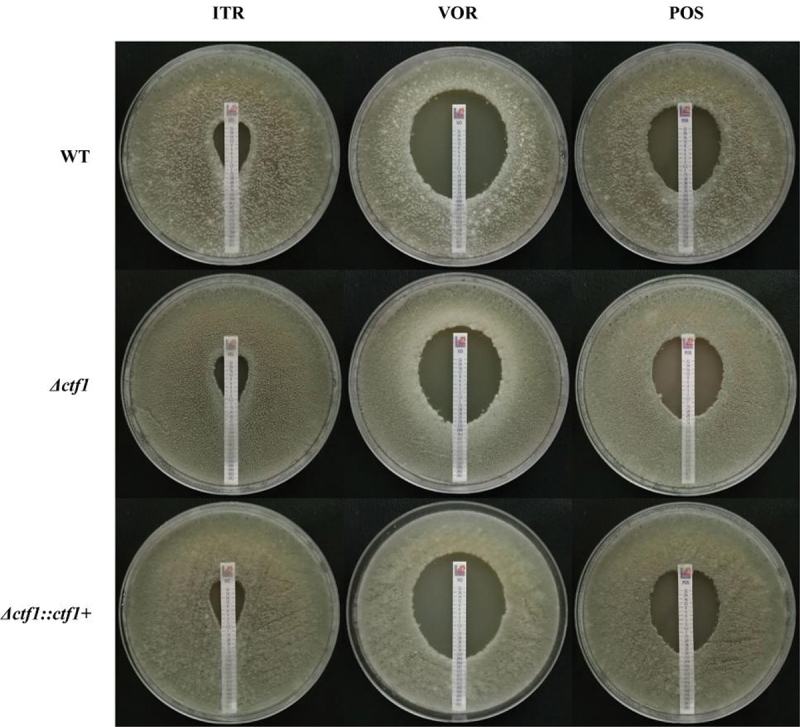
E-test. Each of the three strains was incubated in 1640 solid medium containing azole reagent strips for 48 h at 35 °C. The *ctf1* gene-deficient strains displayed decreased sensitivity to VOR, but no significant change in sensitivity to ITR and POS.

E-test and the micro-liquid-based dilution method were performed to investigate the role of ctf1 in antifungal sensitivity. *Δctf1* displayed decreased sensitivity towards voriconazole (VOR), whereas no significant change was found in sensitivity towards itraconazole (ITR) and posaconazole (POS) (Figure 3, Table 1). *Δctf1∷ctf1+* exhibited the same azole sensitivity compared with WT. The results of these two experiments confirmed that *ctf1* plays a role in the sensitivity of *A. fumigatus* towards VOR. Furthermore, the *ctf1* mutation decreased the sensitivity of *A. fumigatus* towards VOR.

**Table 1.** Effect of *ctf1* on the susceptibility of triazole antifungal drugs *in vitro*.

Strains	Agent alone MIC^a^ (μg/mL)					
	Via liquid-based dilution drug sensitivity test			Via E-test		
	ITR	VOR	POS	ITR	VOR	POS
WT	1	0.25	0.5	1.5	0.094	0.19
*Δctf1*	1	1	0.5	2	0.19	0.25
*Δctf1::ctf1+*	1	0.25	0.5	1.5	0.094	0.19

^a^The MIC is the concentration achieving 100% growth inhibition for *Aspergillus*.

### 3.4. *Response of differentially expressed genes (DEGs) to* ctf1 *defects*

**Figure 4.**
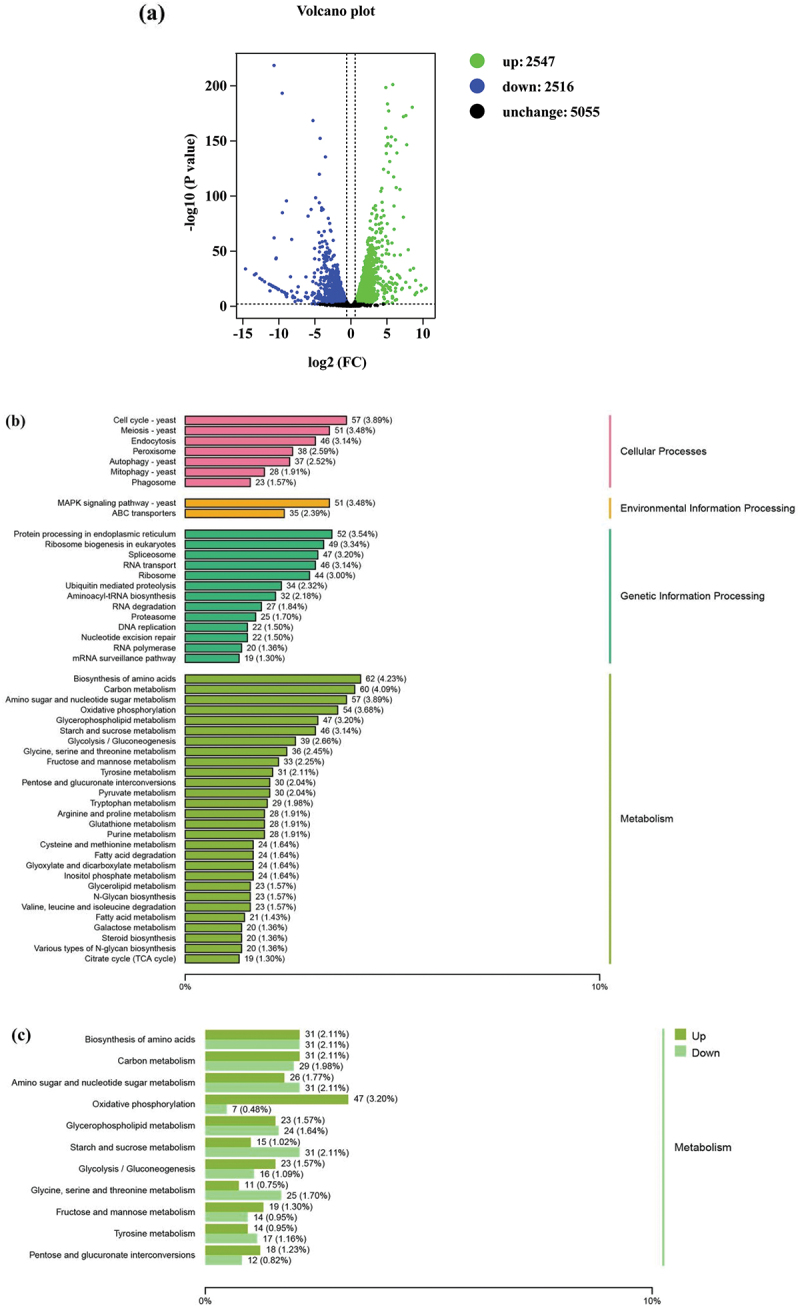
Analysis of DEGs in WT and *Δctf1* transcriptomes. (a) Volcano plot of all detected genes. The transcriptome results identified 2,547 genes with upregulated expression and 2,516 genes with downregulated expression. (b) KEGG pathway analysis of DEGs showed that 35 DEGs were mainly involved in “ABC transporters”. Most genes (61.57%) affected by *ctf1* were associated with fungal metabolism. (c) The expression of differential genes in pathways associated with metabolism.

We performed RNA transcriptome sequencing analyses to characterise the function of *ctf1* in *A. fumigatus*. In total, 5,063 genes were differentially expressed in the WT and *Δctf1* groups, of which 2,547 (48%) and 2,516 (52%) were upregulated and downregulated, respectively (Figure 4(a)). Kyoto Encyclopedia of Genes and Genome (KEGG) pathway analysis of these differential genes indicated that *ctf1* affected the cell cycle of *A. fumigatus* (Figure 4(b)), thereby confirming that *ctf1* influences the growth of *A. fumigatus*. In total, 35 (2.39%) differential genes were involved in the processing of environmental information of ABC transporter proteins, which can be related to the molecular mechanism through which *ctf1* affects the sensitivity of *A. fumigatus* towards azoles. In addition, 61.57% of the differential genes were mainly involved in the pathways of primary metabolite metabolism, oxidative phosphorylation, and secondary metabolite metabolism. The majority of sugar metabolism-related genes demonstrated downregulation after *ctf1* deletion, whereas 47 out of 54 oxidative phosphorylation-related differential genes showed upregulation (Figure 4(c)), which may have altered the focus of fungal energy metabolism. Therefore, these metabolic activities play an indispensable role in response to *ctf1* mutations.

### 3.5. *Effect of* ctf1 *on the expression of* A. fumigatus *antifungal resistance-related genes*

**Figure 5.**
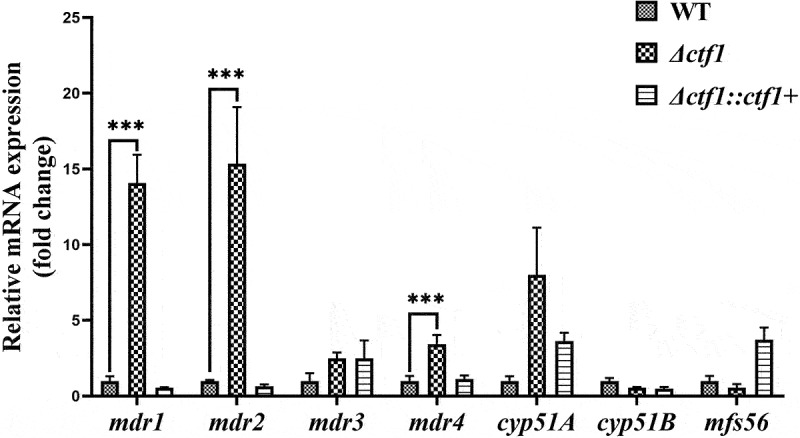
qPCR analysis. The expression levels of *mdr1*, *mdr2*, and *mdr4* were significantly elevated in *Δctf1* compared to WT. Data were analysed by using the 2^−ΔΔCT^ method. ***p < 0.001.

To elucidate the mechanism underlying the decreased sensitivity of *Δctf1* towards VOR, we used real-time quantitative polymerase chain reaction (PCR) to evaluate the expression of *A. fumigatus mdr1*, *mdr2*, *mdr3*, *mdr4*, *cyp51A*, *cyp51B*, and *mfs56*. We found that the expression of *mdr1*, *mdr2*, and *mdr4* was significantly increased in *Δctf1*, while the expression of *mdr3*, *cyp51A*, *cyp51B*, and *mfs56* was not significantly different when compared with that in WT (Figure 5).

### 3.6. *Effect of* ctf1 *on ergosterol synthesis in* A. fumigatus

**Figure 6.**
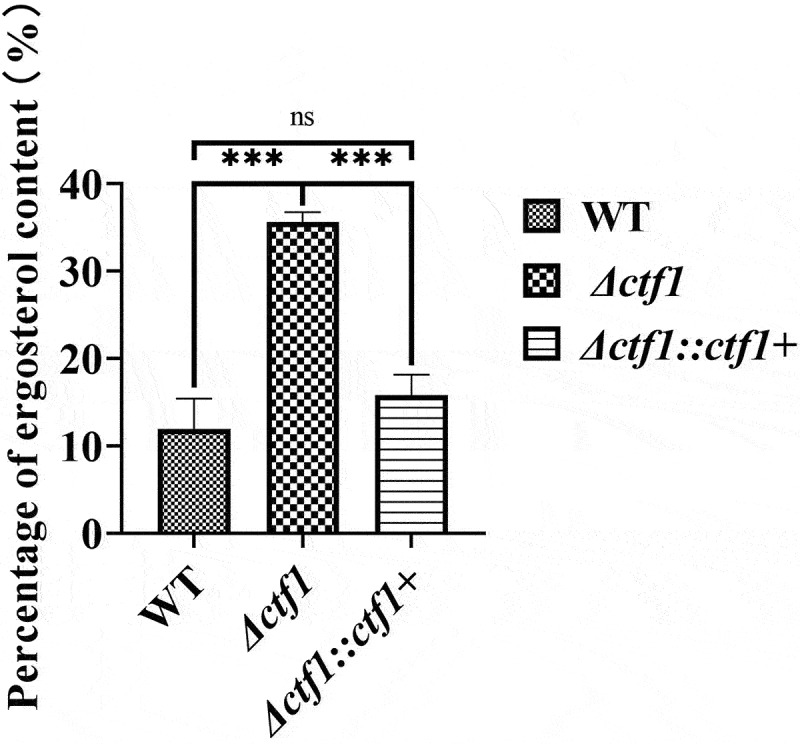
Comparison of the ergosterol content in the three strains. Colonies in SDA dishes after incubation at 37 °C for 72 h. The sterols of the fungus were extracted with n-heptane. The absorbance at 240 nm to 300 nm was measured and the ergosterol content was quantified by using the formula (Percentage of ergosterol content $\left(\%\right)=\left\{\left[\left(A281.5/290\right)\;\times F]/\mathrm{wet}\;\mathrm{weight}\text{\textbackslash{}break}-[\left(A230/518\right)\;\times F\right]/\mathrm{wet}\;\mathrm{weight}\right\}\;\times \;100\%$). The ergosterol content of the knockout strain was significantly higher compared to WT and was largely restored after reintroduction of the *ctf1*. ***p < 0.001; ns, *p* > 0.05.

The ergosterol content of the three strains was determined. The ergosterol content of *A. fumigatus* was significantly increased when the *ctf1* was mutated, and the ergosterol content was restored after the *ctf1* was restored (Figure 6). This implies that *ctf1* correlates with ergosterol content in *A. fumigatus*.

## 4. Discussion

C6 transcription factors are detected only in fungi and are functionally diverse. They are involved in the regulation of *Aspergillus* growth and development, biosynthesis of secondary metabolites, oxidative stress resistance, and virulence (Fernandes et al. 1998; Nascimento et al. 2003; Yin et al. 2012; Ries et al. 2020; Zhao et al. 2022), and they can positively or negatively regulate gene expression and influence cellular functions (MacPherson et al. 2006). In this study, we identified a novel C6 transcription factor, *ctf1*; this gene had no functional annotation earlier. It regulates the strains’ growth, spore production, ergosterol synthesis, and azole sensitivity in *A. fumigatus*.

Invasive aspergillosis can affect immunocompromised individuals. Conidia are the most important pathogens of these infections. They are small and hydrophobic and can easily disperse into the air and survive under harsh environmental conditions (Dagenais and Keller 2009). Our experimental results indicated that *ctf1* knockdown could inhibit the growth of *A. fumigatus* and decrease its conidial production, implying that *ctf1* may be involved in regulating the pathogenicity of *A. fumigatus* by affecting growth and development. By analysing the DEGs of the RNA transcriptomes, we found that *ctf1* regulates the cell cycle, which may be the reason why *ctf1* mutation leads to the inhibition of growth and spore production in *A. fumigatus*.

Currently, triazoles are the antifungal drugs of choice. The emergence of azole-resistant strains has challenged clinical treatment. So far, the mechanism of azole resistance has not been fully investigated, and it is recognised as a complex event influenced by multiple factors, including alterations in drug targets, loss in drug efficacy, and metabolic bypass (Shishodia et al. 2019). We found that *ctf1* gene-mutated strains of *A. fumigatus* exhibited a 2-fold decrease in susceptibility towards VOR compared with WT. On the other hand, the revertant strains displayed the same susceptibility towards VOR when compared with WT strains, indicating that *ctf1* deletion, which is required for the maintenance of normal VOR resistance, is responsible for this phenotype. Triazoles primarily inhibit Cyp51, which is a major enzyme in the ergosterol biosynthesis pathway of *Aspergillus*. This event leads to the accumulation of 14-methylated sterols, which then induces changes in the fluidity of the fungal cell membranes and eventually leads to its disruption. This event decreases the activity of the membrane-bound enzymes and inhibits cell growth and proliferation (Pérez-Cantero et al. 2020). The most common mechanism of azole resistance is inducing changes in *cyp51A*. However, our qPCR result displayed no significant alterations in the expression of *cyp51A* and *cyp51B* in the knockout strain, suggesting that the decreased VOR sensitivity caused by *ctf1* deletion does not appear to be related to *cyp51*. Similar data were observed in a recent research on the mechanism of azole resistance in *Aspergillus* section *Nigri*, suggesting that the *cyp51* gene has a limited/no role in azole resistance, whereas upregulation of the *mdr* genes contributing resistance against azole (Sen et al. 2024).

Several studies have demonstrated that the overexpression of efflux pumps is associated with azole resistance in *A. fumigatus* biofilms (Coleman et al. 2009), including ABCs and major facilitator superfamily (MFS) transporter proteins. These two proteins possess very different structures and action mechanisms. For instance, ABC transporter proteins consist of two transmembrane structural domains and two cytoplasmic nucleotide-binding structural domains and use the energy generated by ATP hydrolysis to efflux the substrate across the membrane, whereas the MFS transporter contains 12–14 transmembranal structural domains and uses proton motive force to efflux the drug in almost all cases (Law et al. 2008; Perlin et al. 2015). In most cases, the overexpression of these transporter proteins may affect the intracellular drug levels such that it does not reach the levels necessary to be effective against fungal cells (Perlin et al. 2015). The KEGG pathway analysis revealed that *ctf1* did affect ABC transporter protein environmental information processing. The qPCR result indicated a significant increase in *mdr1*, *mdr2*, and *mdr4* in *Δctf1*, and a restoration in the expression of these genes in *Δctf1∷ctf1+*. Previous studies reported that the ABC transporter proteins *mdr1*, *mdr2*, and *mdr4*, and the MFS transporter protein *mdr3* are associated with increased azole resistance (da Silva Ferreira et al. 2004; Paul et al. 2017; Sen et al. 2023). The decreased VOR sensitivity due to *ctf1* mutations in this experiment can most likely be attributed to increased drug efflux by the overexpression of *mdr1*, *mdr2*, and *mdr4*, which is not related to *mdr3* and *mfs56*. The overexpression of these transporter proteins influences the intracellular drug levels such that they do not reach the levels necessary to be effective against fungal cells (Coleman et al. 2009). Thus, the *ctf1* is probably important for maintaining the expression of *mdr1*, *mdr2*, and *mdr4*.

Biofilms are structured groups of microbial cells attached to the surface of fungi and are also believed to be associated with fungal drug resistance. The resistance of *A. fumigatus* biofilms to voriconazole increases with biofilm maturation (Mowat et al. 2008). We found that *ctf1* predominantly influences growth and metabolic processes in *A. fumigatus*, with *ctf1* defect downregulating glucose metabolism-related genes and upregulating oxidative phosphorylation-related genes. It has been shown that early biofilms are energy-dependent, whereas mature biofilms are characterised by a reduction in metabolic activity, which is associated with the downregulation of glycolysis/gluconeogenesis and the tricarboxylic acid cycle (Gibbons et al. 2012; Muszkieta et al. 2013), and biofilms mature not through fermentation but through oxidative phosphorylation for energy. The *mdr4* is upregulated in a biofilm phenotype (Nascimento et al. 2003). We thus hypothesised that *ctf1* is associated with cellular biofilm maturation.

Furthermore, we found that the ergosterol content in *Δctf1* was significantly increased when compared with WT and *Δctf1::ctf1+*, which may have improved the stability of fungal cell membranes, thus making the fungus less sensitive towards azoles. *A. fumigatus* can uptake exogenous cholesterol under aerobic conditions, thereby inhibiting the antifungal efficacy of sterol biosynthesis inhibitors (Xiong et al. 2005). Recent findings on AtrR suggest that this C6 transcription factor plays a key role in novel azole resistance mechanisms via co-regulating drug targets (Cyp51A) and putative drug efflux pumps (Cdr1B) (Hagiwara et al. 2017). Our study found that *ctf1* does not affect the ergosterol content through the overexpression of *cyp51A* and *cyp51B*. Mitochondria act as energy factories, producing about 90% of the chemical energy needed by the cell (Henze and Martin 2003). Mitochondrial inhibition leads to a decrease in the conversion of 14-α-methylfetosterol to 14-methylsterols, accompanied by an increase in the conversion of the former to episterol, which leads to the increased formation and more preservation of ergosterol under the influence of Erg3p (Kontoyiannis 2000). In mitochondria, the activity of the mitochondrial complex plays a key role in electron transport and oxidative phosphorylation, which is also a major factor in the production of reactive oxygen species (ROS) by the cell (Murphy 2009). The mechanism of non-*cp51A* mutation-mediated *Aspergillus* resistance was found to be due to azole antifungals reducing ROS production via decreasing the activity of the mitochondrial complex enzymes (Zhu et al. 2023).

In this study, we first characterised a novel C6 transcription factor called *ctf1*, which is an important regulator of the expression of growth, metabolism, and efflux pumps of *A. fumigatus. ctf1* needs to maintain the normal voriconazole sensitivity of *A. fumigatus*. The study of the function of *ctf1* will be an important contribution to the elucidation of the mechanism of *A. fumigatus* resistance and of the *A. fumigatus* virulence-associated factors.

## Supplementary Material

Supplemental Material
