# Correction

**DOI:** 10.1080/21501203.2025.2461912

**Published:** 2025-02-06

**Article title**: Aspergillus fumigatus ctf1–a novel zinc finger transcription factor involved in azole resistance

**Authors**: X. Gong., H. Zhang., W. Cheng., Z. He., T. Ma., T. Chen., & Y. Sun

**Journal**: *Mycology*

**DOI**: https://doi.org/10.1080/21501203.2024.2342521

This article was previously with an error in table. This has now been added and republished in the original article.

**Table 1.** Effect of ctf1 on the susceptibility of triazole antifungal drugs *in vitro*.

Strains	Agent alone MIC^a^ (μg/mL)						
	Via liquid-based dilution drug sensitivity test				Via E-test		
	ITR	VOR	POS		ITR	VOR	POS
WT	1	0.25	0.5		1.5	0.094	0.19
*Δctf1*	1	1	0.5		2	0.19	0.25
*Δctf1∷ctf1+*	1	0.25	0.5		1.5	0.094	0.19

^a^ The MIC is the concentration achieving 100% growth inhibition for *Aspergillus*

