# Supplementary material for: Aspergillus fumigatus ctf1–a novel zinc finger transcription factor involved in azole resistance
Source: Mycology. 2024 Apr 23;16(1):266–79. doi: 10.1080/21501203.2024.2342521 (PMC11899207; doi:10.1080/21501203.2024.2342521)
Supplement: Supplemental Material [file TMYC_A_2342521_SM5438.docx]

**Supplemental Table 1.** Detailed information on the primer sequences used in the PCR experiments.

| Primer name | Primer sequence (5'→3') |  |
| --- | --- | --- |
| P1 | TGACACTCGTACTGACGCCCCTTGA | Amplify the upstream of *ctf1* |
| P2 | TAGTTCTGTTACCGAGCCGGTGACGAACGATGGGGAATGGCTGAG | Amplify the upstream of *ctf1* |
| P3 | GCTCTGAACGATATGCTCCAACATCAGCGTGTTACGTCGAATCTCG | Amplify the downstream of *ctf1* |
| P4 | TCCACCGCCGCAAATCGCGCATTCT | Amplify the downstream of *ctf1* |
| pyrG-n-F | CCGGCTCGGTAACAGAACTACCGCAGACAATGCTCTCTATC | Amplify *pyrG* |
| pyrG-n-R | GTTGGAGCATATCGTTCAGAGCAATACCGTTACACATTTCCA | Amplify *pyrG* |
| P5 | CGCGACATTCGAATATCACTGTGGT | Fusion PCR |
| P6 | TACGCAATAGAGGAATTGCACAGCG | Fusion PCR |
| Awm-F1 | CCTCGCACAGACAACCAAG | Verify *pyrG* is inserted into the knockout strain |
| Awm-F2 | ACAAATAGCTGATGCGTAGTGA | Verify *pyrG* is inserted into the knockout strain |
| ctf1-F | CTCGAGGGGGGGCCCGGCTGAACCTAATTCGTGGAGT | Amplify *ctf1* |
| ctf1-R | TCTCGCCACGTTCGCCCTTTGTAACAGAGTATAAGATGTGGG | Amplify *ctf1* |
| ctf1-yz-F | CATCTCCGTCTTCGTCAAGTAA | Verify the *ctf1* and *hph* is present in the reply plasmid |
| ctf1-yz-R | ACAGCATCAGGCTCTGCGTAT | Verify the *ctf1* and *hph* is present in the reply plasmid |
| hph-F | ATCGTTATGTTTATCGGCACTTTG | Verify *hph* is inserted into the reply strain |
| hph-R | TGTTGGCGACCTCGTATTGG | Verify *hph* is inserted into the reply strain |

The underlined sequences are complementary linkage fragments required for fusion PCR.

**Supplemental Table 2.** Detailed information on the primer sequences used in the qPCR experiments.

| Primer name | Primer sequence (5'→3') |
| --- | --- |
| Actin F | GCACGTGAAATTGTTGAAAGG |
| Actin R | CAGGCTGGCCGCATTG |
| *mdr1*-F | TTCCCTTGTTCACAATTCTCTTCG |
| *mdr1*-R | TGACATAGACTGTGACAAACTCG |
| *mdr2*-F | TTTAGCTCCACCGGGTTTG |
| *mdr2*-R | TCGAAAGACCGAACATGCTTGA |
| *mdr3*-F | TCTGATGGCGGTCATCACT |
| *mdr*3-R | ATATCCATCCCCCAGGC |
| *mdr4*-F | TATGGCTTAGTTTGTTTGTGTCACCGA |
| *mdr4*-R | AGAGCAATTCGTTGCTTCTG |
| *cyp51A*-F | TGCAGAGAAAAGTATGGCGA |
| *cyp51A*-R | CGCATTGACATCCTTGAGC |
| *cyp51B*-F | AGCAGAAGAAGTTCGTCAAATAC |
| *cyp51B*-R | TCGAAGACGCCCTTGTG |
| *mfs56*-F | GGGGGTATGGTAATTGGAGGT |
| *mfs56*-R | AAGAAGCGCAGACCATCG |

F: Forward primer; R: Reverse primer.
